# Model-Independent 3D Descriptors of Vertebral Cancellous Bone Architecture

**DOI:** 10.4061/2010/641578

**Published:** 2009-12-31

**Authors:** Ian H. Parkinson, Danielle Forbes, Peter Sutton-Smith, Nicola L. Fazzalari

**Affiliations:** Bone and Joint Research Laboratory, SA Pathology and Hanson Institute, Discipline of Pathology, University of Adelaide, Adelaide, South Australia 5000, Australia

## Abstract

High-resolution micro computed tomography has enabled measurement of bone architecture derived from 3D representations of cancellous bone. Twenty-eight vertebral bodies were obtained from four embalmed male cadavers. From 3D anaglyphs, trabecular rod thickness and length were measured and the trabecular rod Buckling index was calculated. From 3D voxel-based datasets, bone volume density, trabecular thickness, and trabecular separation were measured. Also, trabecular bone pattern factor, structural model index, connectivity density, and degree of anisotropy were calculated. Bone volume density alone explains 59% of the variability in trabecular rod Buckling index. The addition of connectivity density, trabecular separation, and structural model index, in a multiple regression statistical model, improves the explanatory power to 77%. The relationships between measures of cancellous bone architecture and a derived measure of trabecular rod strength were investigated. Morphological descriptors of cancellous bone provide a composite explanatory model of trabecular rod strength.

## 1. Introduction

The characterisation of bone microarchitecture has been essential in elucidating the pathogenesis of osteoporosis, and alterations in bone microarchitecture have been recognised as playing an important role in the susceptibility of bone to fracture [1, 2]. The ability to quantify the degree to which measures of bone microarchitecture predict bone strength, ex vivo, has provided valuable insights into the mechanisms of fragility fractures. 

The advent of high-resolution micro-computed tomography (micro-CT) and its increasing accessibility to researchers have ushered in a new era in bone histomorphometry. The constraints of extrapolation from two-dimension (2D) to three-dimension (3D) have now been broken enabling the measurement of bone quality indicators derived from accurate 3D voxel-based representations of cancellous bone structure. 

The classic model-based histomorphometry espoused by Parfitt [3, 4] and universally adopted can now be applied to 3D datasets with or without correction factors related to 2D histological sections. However, the model is a simplified representation of the complex cancellous bone structure. Vijayapalan et al. [5] have shown by direct measurement of trabecular rod thickness from 3D anaglyphs that the “true” dimensions of trabecular rods are different to trabecular thickness derived from Parfitt's model. This means that the magnitude of derived dimensions such as trabecular thickness and trabecular separation may not be accurate measures but were useful for demonstrating relative differences between study groups or tracking temporal changes within a study series. 

There have been numerous protocols published describing the adaptation of three-dimensional descriptors of cancellous bone architecture for use with two-dimensional histological sections [6–10]. These include connectivity density, degree of anisotropy, and trabecular bone pattern factor but their utility has been limited because of large systematic errors related to the extrapolation from 2D to 3D [11]. The accurate utilisation of these 3D descriptors of cancellous bone structure has been possible with micro-CT datasets. 

Micro-CT imaging provides a series of 2D tomographs, which enable accurate reconstruction of the specimen as a voxel-based dataset. Each tomograph is equivalent to a histological section, whereby in 2D the bone matrix is clearly delineated from marrow spaces. Importantly, the series of tomographs enable an accurate 3D representation of the bone to be constructed. It is possible from this voxel-based dataset to measure trabecular dimensions using a sphere-fitting algorithm [12] and to apply other model-independent algorithms for bone structure, such as connectivity density [11], structural model index [13], and degree of anisotropy [14]. Datasets derived from micro-CT imaging provide a comprehensive suite of descriptive parameters for bone structure in 3D as well as accurate measurement of the amount of bone. 

The increased focus on the functional properties of cancellous bone has highlighted the need to obtain accurate measurements of the dimensions of trabecular elements. It has been shown, theoretically, that the magnitude and variability of trabecular dimensions affect the functional properties of the cancellous structure [15] and that trabecular rods may be the critical structures in determining cancellous bone strength [16]. Measurement of individual trabeculae can be achieved by decomposition of micro-CT datasets ([17, 18], which also enables classification of trabeculae as rods or plates. Recent work has shown that apparent mechanical properties of cancellous bone are compromised, to a greater or lesser degree, when groups of trabeculae with defined spatial orientation are removed in micro-CT derived datasets [18]. Together with location data these algorithms provide the tools to identify the critical structures in cancellous bone that contribute to its strength. 

It has been shown that individual trabeculae fails, in compression, by Euler buckling [16, 19, 20] and Sutton-Smith et al. [21] have shown that using 3D anaglyphs a load to buckling index (Buckling index) can be derived from measurements of trabecular rod thickness and trabecular rod length. It is hypothesised that the distribution of the Buckling index for trabecular rods indicates a potential failure mechanism for the cancellous bone structure. While it has been shown that the distributions of trabecular rod thicknesses and trabecular rod lengths are normally distributed, the distribution of the Buckling index for trabecular rods is a log normal distribution [21]. The shape of this distribution indicates that a force of a critical magnitude will cause a significant number of trabecular rods to fail simultaneously resulting in failure of the entire cancellous structure. 

The aim of this study is to determine the degree to which measures of bone architecture, derived from micro-CT imaging of human vertebral body cancellous bone, explain the variability in a surrogate of bone strength (Buckling index). It is hypothesised that measures of bone architecture in addition to the amount of bone will explain significantly more of the variability in the Buckling index than the amount of bone alone. The degree to which these parameters explain the variability in the Buckling index will be determined using a statistical multiple regression approach. It is expected that the approach used in this study will further identify model-independent descriptors of bone structure, which may determine bone strength.

## 2. Methods

### 2.1. Samples

Thoracolumbar vertebral bodies (T6, T8, T10, T12, L1, L3, and L5) were obtained from four embalmed male cadavers. The donor spines were sourced from a forensic science facility and some vertebral bodies were used in other studies, hence there were not adjacent vertebral bodies. The ages of the cadavers were not known, however an estimate of the age was made using a grading protocol based on the amount of osteophytic lipping [22]. The age estimates were 2 cadavers under 60 years of age and 2 cadavers over 60 years of age. A sagittal slice 2.5-mm in thickness was taken adjacent to the mid-line from all 28 vertebral bodies, which ranged in size from 15 mm to 25 mm crano-caudally to 30 to 40 mm antero-posteriorly. The marrow and adherent soft tissues were removed by maceration. Three-dimensional anaglyphs were obtained by scanning electron microscope (SEM) imaging of nine contiguous fields, each 35 mm^2^ and covering most of the vertebral body slice, at a spatial resolution of 7.83 *μ*m [5]. Each anaglyph was constructed from two digitised SEM images (the second image tilted through 5°) and viewed on a computer screen with red-green stereo glasses.

### 2.2. 3D Anaglyphs

From the 3D anaglyphs, trabecular rod thickness (Tb.Th_(rods)_) and trabecular rod length (Tb.Le_(rods)_) were measured and the load to buckling index (Buckling index) was calculated for a total of 2225 randomly selected trabecular rods (*Buckling index* *r* ^4^/*l* ^2^, *r* = radius of rod and *l* = length of rod). For measurement, the 3D anaglyphs were viewed on a computer screen with red-green stereo glasses using a Quantimet 500MC image analyser (Leica, Cambridge, UK) with a grid at a random angle overlaid to enable random sampling of trabecular rods. The 3D anaglyphs enabled clear identification of trabecular rods as smooth, roughly cylindrical structures, with a length at least three times their width. Measurement of trabecular rod thickness was made at the point of intersection of the rod with the grid and perpendicular with the long axis of the rod [5]. The ends of a trabecula for measuring trabecular length were defined as the midpoints of an arc formed between the trabecular rod and the adjacent trabecular structures [23]. 

### 2.3. Micro-CT

Micro-CT imaging was subsequently performed on the specimens at a spatial resolution of 15.83 *μ*m with a Skyscan 1072 (Skyscan, Kontich, Belgium). Cone-beam reconstruction software yielded up to 100 tomographs for each specimen, in the sagittal plane. Using a custom-written routine in Matlab (MathWorks Ltd, Natick, Mass, USA), a binary image, discriminating bone from marrow in each tomograph, was obtained using Otsu's method [24]. This was followed by a binary closing (One cycle of erode followed by one cycle of dilate), to remove small unconnected artifacts. 

**Figure 1:**
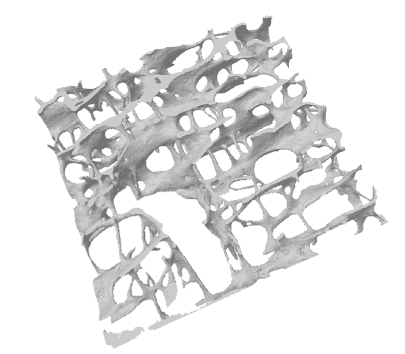
Three-dimensional rendering of voxel-based dataset from a micro-CT scanned sagittal slice of human L1 vertebral body cancellous bone (BV/TV = 9.5%; Tb.Th* = 180 *μ*m; Tb.Sp* = 1037 *μ*m; DA = 11.31; TBPf = 6.4 mm^−1^; Conn.D = 1.14 mm^−1^, and SMI = 1.58). Scanning resolution = 15.63 *μ*m/pixel.

CT analyser software (CTAn) provided by Skyscan uses the marching cubes method to generate a surface rendering of the bone (Figure 1). The volume of interest for each micro-CT dataset was 243 mm^2^.

### 2.4. Mechanical Testing

To establish the efficacy of the Buckling index as an appropriate surrogate of cancellous bone strength, the strength of cancellous bone cubes taken from the centre of 28 vertebral bodies was measured as ultimate failure stress (UFS). The cancellous bone cubes were obtained from the L2 and L3 vertebral bodies of 14 fresh-frozen spines. 

Prior to mechanical testing, micro-CT imaging was performed using the same machine settings as for the sagittal bone slices and which resulted in a micro-CT dataset for each 10 × 10 × 10 mm bone cube consisting of 640 tomographs, which were each 640 pixels by 640 pixels. Trabecular rods were identified using the volumetric spatial decomposition algorithm, where each discrete trabecular element was classified as a trabecular plate or a trabecular rod. The volumetric decomposition algorithm yields length and thickness [17], from which the Buckling index was calculated, as above. Mechanical testing was by uniaxial compression in the supero-inferior direction at 0.1 mm/sec to failure (Hounsfield H25KM, Hounsfield Ltd, UK).

### 2.5. Parameters

Using the 3D voxel-based datasets, the following model-independent parameters were obtained: bone volume per total volume (BV/TV) and trabecular thickness (Tb.Th*) and trabecular separation (Tb.Sp*) using a sphere fitting algorithm [12], trabecular bone pattern factor (TBPf) as the ratio of convex to concave surfaces [8], structural model index (SMI) as an index of how rod-like or plate-like the structure [13], the connectivity density (Conn.D) as an indicator of connectivity within the structure [11], and degree of anisotropy (DA) from mean intercept length analysis as an index of the degree of preferred orientation of the structure [14]. 

From the mechanical testing study, Ultimate failure stress (UFS) was calculated from the stress/strain curve, as a measure of bone strength and the Buckling index was calculated for trabecular rods.

### 2.6. Statistics

For all parameters, a mean value was calculated for each of the 28 vertebral body specimens and used in subsequent statistical analyses. The geometric mean was calculated for the Buckling index from each vertebral body due to the log normal distribution of this parameter. 

The correlations between anaglyph-derived parameters and micro-CT-derived parameters were determined by least-squares linear regression analysis. The degree to which these descriptive parameters explain the variability in the Buckling index was determined by least-squares linear regression analysis. Multiple regression analysis was performed to determine what combination of parameters best explains the variability in trabecular rod Buckling index. This entails sequential addition to the statistical model of measures of bone architecture to determine the best combination of predictors of the Buckling index. 

From the mechanical testing study, the Buckling index for each trabecular rod was calculated as per methods outlined above. The correlation between the Buckling index and UFS was determined by least-squares linear regression analysis. 

All statistical tests were performed using PC-SAS (SAS Institute, Cary, NC). Statistical significance was determined to be *P* < .05. 

## 3. Results

Within the study sample, the vertebral body BV/TV ranged from 8% to 18% with descriptive statistics for all parameters shown in Table 1. 

### 3.1. Correlations between Anaglyph-Derived Parameters and Micro-CT-Derived Parameters

Buckling index significantly correlates with BV/TV, Tb.Th*, Tb.Sp*, TBPf, SMI, and Conn.D (Table 2). It is noteworthy that the correlations of Tb.Th_(rods)_ and Tb.Le_(rods)_ with BV/TV and Conn.D, while statistically significant, are not as strong as the correlations of the Buckling index with BV/TV and Conn.D.

### 3.2. Variability in Buckling Index-Explanatory Models

Least squares linear regression shows that BV/TV explains 59% of the variability in the Buckling index (*r* ^2^ = 0.59; *P* < .0001) (Table 2). Of the other model-independent parameters, Conn.D is the best individual explanatory parameter of the Buckling index (*r* ^2^ = 0.52; *P* < .0001) (Table 2). 

Multiple regression analysis, with *r* ^2^ selection, determines what combinations of parameters explain best the variability in the Buckling index of trabecular rods (Table 3). There is a statistically significant (*P* < .05) improvement in explanatory power for trabecular rod buckling index from 59% of the variability for BV/TV alone to 77% of the variability when Conn.D, Tb.Sp*, and SMI are added to BV/TV in the multiple regression model.

### 3.3. Correlation between the Buckling Index and UFS and Correlation between BV/TV and UFS

**Figure 2:**
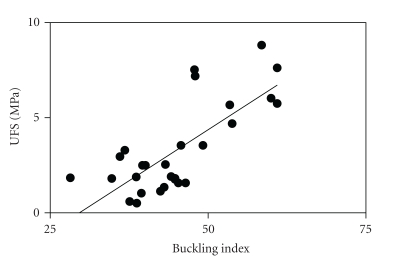
Scatter plot of Buckling index versus UFS (bone strength), showing a statistically significant relationship (*r* ^2^ = 0.56; *P* < .0001).

**Figure 3:**
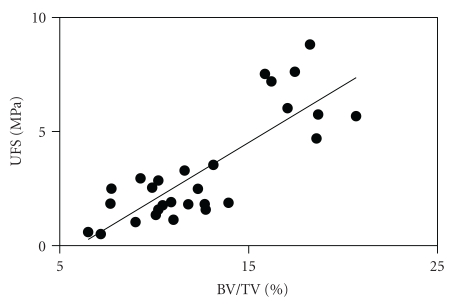
Scatter plot of BV/TV versus UFS (bone strength), showing a statistically significant relationship (*r* ^2^ = 0.68; *P* < .0001).

Buckling index is significantly correlated with UFS (*r* ^2^ = 0.56; *P* < .0001) (Figure 2). BV/TV is significantly correlated with UFS (*r* ^2^ = 0.68; *P* < .0001) (Figure 3).

## 4. Discussion

This study showed that measures of bone architecture, derived from micro-CT imaging of human vertebral body cancellous bone, can explain a statistically significant proportion of the variability in a surrogate of bone strength (Buckling index). The data supports the hypothesis that measures of bone architecture in addition to the amount of bone explain significantly more of the variability in the Buckling index than the amount of bone alone. 

The study sample consisted of vertebral bodies, where BV/TV of the vertebral body cancellous bone encompasses the range found in healthy adults (8% to 18%). Using a 3D-based direct method of measuring trabecular rod dimensions, a surrogate of trabecular rod strength was derived, the Buckling index. Rendering of the tomographs into a 3D voxel-based dataset enables the application of algorithms, which describe model-independent 3D structural characteristics of the cancellous bone (BV/TV, Tb.Th*, Tb.Sp*, TBPf, SMI, Conn.D, and DA). 

The analysis of anaglyphs enables both trabecular rod thickness and trabecular rod length measurement and the derivation of a functional descriptor of the trabeculae, the Buckling index. It is assumed that buckling is the most likely mode of failure for trabecular rods given that they experience predominantly compressive loads and are fixed at both ends [16]. Also, this index of trabecular rod buckling strength is calculated in the direction of the trabecular long axis. To date, it has not been possible to directly measure the strength of individual trabeculae in an intact trabecular network, although the strength of isolated trabeculae has been measured [19, 25]. The strong statistical relationship between the Buckling index and BV/TV translates to a coefficient of determination (*r* ^2^) of 59%, which is within the range of previously published data showing the ability of the amount of bone to explain the variability in vertebral body strength [26]. While the correlations of Tb.Th_(rods)_ and Tb.Le_(rods)_ with BV/TV and Conn.D are statistically significant, they are not as strong as the correlations of the Buckling index with BV/TV and Conn.D. 

The parameters derived from the 3D voxel-based dataset show that individually TBPf and Conn.D correlate strongly with the Buckling index but not with each other. This indicates that the form of individual trabeculae and the connectivity of the bone structure are independently important in determining the strength of the vertebral body. These model-independent algorithms when applied to histological sections were notoriously unreliable because the assumptions made to extrapolate from 2D to 3D were sources of large systematic errors [27]. Micro-CT imaging allows application of the theory underpinning these algorithms with greatly reduced systematic errors. 

The multiple regression analysis shows that the amount of variability in the Buckling index attributable to BV/TV can be improved from 59% to 77% with the addition of model-independent 3D morphological parameters (Conn.D, Tb.Sp*, and SMI). These 3D microarchitecture-related factors explain a further 18% of the variability in the predicted buckling strength of trabecular rods. Addition of more morphological parameters to the statistical model does not significantly improve the prediction of the Buckling index. This statistical model quantifies the contribution of the 3D architecture of the cancellous bone to trabecular rod strength in addition to the contribution of the amount of bone. 

This study identifies important morphological determinants of the mechanical properties of cancellous bone. Specifically, connectivity of the trabecular structure, as measured by Conn.D, would be expected to be an important determinant of structural integrity. In fact Conn.D is almost as strong as an independent determinant of the Buckling index as BV/TV (*r* ^2^ = 0.52 versus *r* ^2^ = 0.59, resp.). Measures of trabecular form and structure such as SMI and TBPf contribute significantly to prediction of the Buckling index, and individually they are both negatively correlated with the Buckling index. This supports the view that more rod-like structures are weaker than more plate-like structures. The degree of anisotropy (DA) does not contribute to the Buckling index because it is a measure of orientation of the entire cancellous structure, whereas the Buckling index of individual trabeculae is orientation independent. Thomsen et al. [28] report a statistically significant improvement in the prediction of bone strength at L3 from 74% to 79% when 2D histomorphometric indices measured at L2, such as Tb.Th, Tb.Sp, and star volume, were added to BV/TV. Hence, bone strength can be more reliably predicted when the contribution of 3D-bone structure is added to the contribution of BV/TV alone. 

A limitation of this study is that there are multiple vertebral levels from a small number of individuals. This study made use of human cadaveric material that was sourced for other studies but was used in this study to maximize the use of this difficult-to-access material. The use of multiple vertebral levels from each individual reduces the statistical power if conclusions as to an individual's risk of fracture were made but in this cross-sectional study where each vertebral body is an independent observation, it is appropriate to make conclusions as to the ability to explain the variability in bone strength within the group of vertebral bodies. Another limitation of this study is that mechanical testing was not performed on the sagittal slices. However, the use of the Buckling index as a surrogate of bone strength provides a morphological framework by which functional aspects of cancellous bone structure can be studied. Specimen handling or processing prior to micro-CT imaging can compromise the ability to perform mechanical testing. Other recent studies have quantified the role of microarchitecture [29] and the effect of regional variability [30] in determining vertebral body cancellous bone strength and whilst these studies have utilised mechanical testing as the outcome variable, their results are not inconsistent with the results of this study. Namely, that cancellous bone *per se* is an important determinant of vertebral body strength and that the contribution of microarchitecture of individual trabeculae to bone strength can be quantified. Therefore, a purely morphological approach, such as the one presented in this study can provide useful data on the functional characteristics of cancellous bone. 

In this study, the morphological descriptors of vertebral body cancellous bone derived from micro-CT images provide a composite explanatory model of trabecular rod strength (Buckling index). The literature does not provide many theoretical alternatives to the Buckling index as a descriptor of the functional properties of trabeculae. While in this study a relatively small sample of vertebral bodies are studied, the strength of the relationships outlined above give grounds to further investigate the use of a functional descriptor of cancellous bone derived from 3D morphological parameters of cancellous bone.

## Figures and Tables

**Table 1:** Descriptive statistics for all parameters (mean ± sd).

		Mean ± sd
Micro-CT derived	BV/TV (%)	16.54 ± 4.96
	Tb.Th* (*μ*m)	172 ± 19
	Tb.Sp* (*μ*m)	681 ± 194
	TBPf (mm^−1^)	6.92 ± 1.09
	SMI	1.59 ± 0.23
	Conn.D	6.10 ± 4.59
	DA	10.53 ± 1.73
Anaglyph derived	Tb.Th_(rods)_ (*μ*m)	143 ± 17
	Tb.Le_(rods)_ (*μ*m)	576 ± 68
		Geometric mean ± sd

	Buckling index (*μ*m^2^)	1000 ± 341

**Table 2:** Statistical correlations between micro-CT-derived parameters and anaglyph-derived parameters (*P < .05; **P < .01; ***P < .001).

	Tb.Th_(rods)_	Tb.Le_(rods)_	Buckling index
BV/TV	0.28**	0.28*	0.59***
Tb.Th*	0.09	0.42**	0.15*
Tb.Sp*	0.06	0.60***	0.36**
TBPf	0.39**	0.11	0.23**
SMI	0.44***	0.005	0.49***
Conn.D	0.16*	0.44***	0.52***
DA	0.004	0.0003	0.002

**Table 3:** Multiple regression analysis with *r* ^2^ selection, with Buckling index as the independent variable.

Parameters	*r* ^2^
BV/TV	0.59
BV/TV + SMI	0.63
BV/TV + Conn.D + Tb.Sp*	0.76
BV/TV + Conn.D + Tb.Sp* + SMI	0.77
